# The Cardioprotective Effects of Citric Acid and L-Malic Acid on Myocardial Ischemia/Reperfusion Injury

**DOI:** 10.1155/2013/820695

**Published:** 2013-05-14

**Authors:** Xilan Tang, Jianxun Liu, Wei Dong, Peng Li, Lei Li, Chengren Lin, Yongqiu Zheng, Jincai Hou, Dan Li

**Affiliations:** ^1^Experimental Research Center, Xiyuan Hospital, China Academy of Chinese Medical Sciences, Beijing 100091, China; ^2^Key Laboratory of Modern Preparation of TCM, Jiangxi University of Traditional Chinese Medicine, Nanchang 330004, China; ^3^Beijing University of Chinese Medicine, Beijing 100029, China

## Abstract

Organic acids in Chinese herbs, the long-neglected components, have been reported to possess antioxidant, anti-inflammatory, and antiplatelet aggregation activities; thus they may have potentially protective effect on ischemic heart disease. Therefore, this study aims to investigate the protective effects of two organic acids, that is, citric acid and L-malic acid, which are the main components of *Fructus Choerospondiatis*, on myocardial ischemia/reperfusion injury and the underlying mechanisms. In *in vivo* rat model of myocardial ischemia/reperfusion injury, we found that treatments with citric acid and L-malic acid significantly reduced myocardial infarct size, serum levels of TNF-*α*, and platelet aggregation. *In vitro* experiments revealed that both citric acid and L-malic acid significantly reduced LDH release, decreased apoptotic rate, downregulated the expression of cleaved caspase-3, and upregulated the expression of phosphorylated Akt in primary neonatal rat cardiomyocytes subjected to hypoxia/reoxygenation injury. These results suggest that both citric acid and L-malic acid have protective effects on myocardial ischemia/reperfusion injury; the underlying mechanism may be related to their anti-inflammatory, antiplatelet aggregation and direct cardiomyocyte protective effects. These results also demonstrate that organic acids, besides flavonoids, may also be the major active ingredient of *Fructus Choerospondiatis* responsible for its cardioprotective effects and should be attached great importance in the therapy of ischemic heart disease.

## 1. Introduction

Ischemic heart disease is a leading cause of mortality of the clinical cardiovascular diseases and remains a major public health threat worldwide. Myocardial damage in ischemic heart disease is likely due to ischemia/reperfusion injury. Myocardial ischemia/reperfusion can lead to cardiomyocyte loss by several pathological mechanisms, which contain free radical formation, inflammatory response and endothelial dysfunction, platelet aggregation and microembolization, necrosis and apoptosis, and so forth [1]. Therefore, a pharmacologic approach to ischemia/reperfusion injury remains a longstanding challenge in medicine.

*Fructus Choerospondiatis*, a widely known Mongolian herb derived from the dried mature fruit of *Choerospondias axillaris *(Roxb.) Burtt *et* Hill, with efficacy of “activating vital energy and blood circulation” and “nourishing heart for tranquilization” [2], according to traditional Chinese medicine theory, has been used extensively as a remedy for ischemic heart disease and achieved good clinical efficacy. It consists of several ingredients, including organic acids, phenolic acids, and flavonoids. Previous studies have been focused on flavonoids, which were always considered to be the main active constituents responsible for the pharmacological actions of *Fructus Choerospondiatis* [3]. However, recent pharmaceutical chemistry studies showed that the content of total flavonoids (mainly quercetin) in *Fructus Choerospondiatis* was very low and only accounted for 0.0003% of its water-soluble extracts [4], whereas the content of total organic acids was in significant amounts, up to 8.13%. And citric acid and L-malic acid are two main organic acids of* Fructus Choerospondiatis*, the content of which accounted for 26.36% and 22.95% of total organic acids, respectively [5].

Organic acids, which are also widely distributed in fresh fruits, vegetables, and spices besides Chinese herbs, were always considered to be weak in activity and were usually discarded in the extraction process. Therefore, they have been long neglected and their pharmacological actions have not been sufficiently studied. Recent research indicated that some organic acids have various pharmacological effects, including anti-inflammatory response [6, 7], antiplatelet aggregation [8], antioxidant [9, 10], and reducing cell apoptosis and so on. Therefore, we hypothesized that organic acids might have protective effect on myocardial ischemia/reperfusion injury.

In the present study, we investigated the protective effects of citric acid and L-malic acid on myocardial ischemia/reperfusion and its possible mechanisms. To the best of our knowledge, the finding that citric acid and L-malic acid have protective effects on myocardial ischemia/reperfusion injury by anti-inflammatory, antiplatelet aggregation, and direct cardiomyocyte protective effects is therefore particularly significant for providing important insights into the understanding of cardioprotective effects of organic acids in traditional Chinese medicine.

## 2. Materials and Methods

### 2.1. Animals

Male adult Wistar rats (210–240 g body weight) were provided by the Experimental Animal Research Institute, Chinese Academy of Medical Sciences (clean degree, certificate no. SCXK (Jing) 2009-0007). Neonatal Sprague-Dawley rats (SPF degree, 1 to 2 days old) were purchased from Beijing Vital River Laboratory Animal Technology Co.Ltd., China (certificate no. SCXK (Jing) 2012-0001). Rats were housed under standard conditions and supplied with drinking water and food *ad libitum*. All animal experiments in this study were performed in accordance with China Academy of Chinese Medical Sciences Guide for Laboratory Animals that conforms to the Guide for the Care and Use of Laboratory Animals published by the U.S. National Institutes of Health (NIH Publications no. 85-23, revised 1996).

### 2.2. Reagents and Chemicals

Lactate dehydrogenase (LDH) assay kit was purchased from Beijing Zhongsheng Biological Technology Co., Ltd. (batch no. 110391). Rat tumor necrosis factor-*α* (TNF-*α*) Quantikine ELISA kit was obtained from R&D (Catalog no. RTA00, USA). FITC-annexin V/propidium iodide apoptosis detection kit was from BD Biosciences (Catalog no. 556547, USA). Nitroblue tetrazolium (N-BT) (Ultra Pure Grade, 0329) and anti-cleaved caspase-3 antibody (Catalog no. C8487) were products of Sigma Chemical Co. (USA). The antibodies for anti-p-Akt (Ser473, no. 4060) and anti-Akt (no. 9272) were from Cell Signaling (USA). Citric acid (batch no. 111679-200401) and L-malic acid (batch no. 190014-201001) were purchased from National Institutes for Food and Drug Control (Beijing, China). All chemicals used were of analytical grade.

### 2.3. Drug Pretreatment and Myocardial Ischemia/Reperfusion Protocols

The studies of citric acid and L-malic acid on myocardial ischemia/reperfusion were performed independently although the experimental designs of them were identical. All animals were randomly assigned to six groups (*n* = 10 for each group). Vehicle or drugs were fed once daily (10 mL/kg) for 3 consecutive days prior to the experiment and treated as follows.

 Group 1: sham group. Rats were orally administered 0.9% saline.
 Group 2: ischemia/reperfusion (I/R) model control group. Rats were also orally administered 0.9% saline.
 Group 3: diltiazem (Tanabe Seiyaku Co., Ltd., Tianjin)-pretreated group as positive control. Rats were orally administered diltiazem at a dose of 16 mg/kg.
 Group 4: clopidogrel (Sanofi Winthrop Industrie)-pretreated group as another positive control. Rats were orally administered clopidogrel at a dose of 13.5 mg/kg.
 Group 5: citric acid- or L-malic acid-pretreated group. Rats were orally administered citric acid or L-malic acid at a dose of 500 mg/kg.
 Group 6: citric acid- or L-malic acid-pretreated group. Rats were orally administered citric acid or L-malic acid at a dose of 250 mg/kg.

Myocardial ischemia/reperfusion injury rat model was constructed by LAD ligation for 40 min followed by 2 h reperfusion at 1 h after the last drug treatment as previously described [11]. Rats were anesthetized with 3.5% chloral hydrate (Sinopharm Chemical Reagent Co., Ltd., China) (350 mg/kg, i.p.). The neck was opened with a ventral midline incision. The trachea was exposed and cannulated to establish artificial respiration provided by a rodent ventilator (ALC-V8S, China) with oxygen at a breath ratio of 1 : 2 and at a frequency of 70 breaths/min with tidal volume of 9.0 mL. Myocardial ischemia was produced by exteriorizing the heart through a left thoracic incision and placing a 5-0 silk suture and making a plastic tubing at the distal 1/3 of the left anterior descending coronary artery. After 40 min of ischemia, the plastic tubing was cut and the myocardium was reperfused for 2 h.

### 2.4. Measurement of Myocardial Infarct Size

Myocardial infarct size was evaluated by N-BT staining as previously described [12]. Briefly, at the end of 2 h reperfusion, rats were anesthetized with 3.5% chloral hydrate and then sacrificed. The hearts were quickly excised and sliced into 5 sections from the position under the ligation line. The slices were weighed and then immediately incubated in N-BT staining solution dissolved in saline at 25°C for 15 min. The infracted size, noninfracted size, and total heart size were measured by multimedia color pathological image analytical system (MPIAS-500, Beijing). The infarction percentage of the ventricle, infarction percentage of the heart, infarction area, and infarction weight were calculated.

### 2.5. Determination of Inflammatory Cytokine Activity and Platelet Maximum Aggregation Rate

The serum levels of TNF*-*α** were measured using ELISA kits according to the manufacturer's instructions. The absorbance at 450 nm was measured on a microplate reader (BioTek SYNERGY 4, USA).

Blood was collected from the abdominal aorta and anticoagulated with citrate (3.8%, 1 : 9, v/v). Platelet-rich plasma (PRP) was obtained by centrifugation at 1000 rpm at 25°C for 10 min and the remaining blood was further centrifuged at 3000 rpm at 25°C for 10 min to prepare platelet-poor plasma (PPP). The platelet concentration was adjusted to 4 × 10^8^ platelets/mL. The platelet agonist adenosine diphosphate disodium (Shanghai Institute of Biochemistry, Chinese Academy of Sciences) (1 mmol/L, 10 *μ*L) was used to stimulate platelet aggregation. The level of platelet aggregation was measured using an aggregometer (BS634, Beijing) according to the method reported by Born and Cross [13]. 

### 2.6. Cell Culture, Hypoxia/Reoxygenation (H/R), and Drug Treatment

Primary cultures of neonatal rat cardiomyocytes were prepared as previously described with some modifications [14, 15]. In brief, the hearts were removed and washed with cold PBS. The atria and aorta were discarded. The ventricles were minced with scissors into 1 mm^3^ fragments. The tissue fragments were digested by gentle shaking at 37°C in PBS containing 0.625 g/L trypsin (Gibco) and 0.5 g/L collagenase II (Gibco). The digestion was conducted for 5–8 times, 10 min each. The dispersed cells were incubated on a 100 mm culture dish for 1 h at 37°C in a humidified incubator with 5% CO_2_. The nonadherent cells were harvested and then seeded into gelatin-coated 6-well plates and incubated in Dulbecco's modified Eagle's medium (DMEM) (Gibco) with 10% newborn calf serum (TBD21HY, Tianjin), penicillin (100 U/mL), streptomycin (100 U/mL), and 5-bromo-2′-deoxyuridine (0.1 mmol/L, sigma), which was used to inhibit cardiac fibroblasts growth.

Hypoxia/reoxygenation-induced cardiomyocytes injury was performed as described previously [15]. Before hypoxia, cardiomyocytes were washed three times with glucose-free Tyrode's solution (in mmol/L: NaCl 136.9, KCl 2.68, NaH_2_PO_4_ 0.42, NaHCO_3_ 11.9, MgCl_2_ 1.05, and CaCl_2_ 1.8). Cells were incubated with glucose-free Tyrode's solution (2 mL/well) saturated with 95% N_2_ and 5% CO_2_ for 15 min. Cells were placed into a hypoxia chamber which was then ventilated with 95% N_2_ and 5% CO_2_ for 15 min and maintained at 37°C in a humidified incubator with 5% CO_2_ for 3 h. Reoxygenation was accomplished by replacing the glucose-free Tyrode's solution with normal cell medium under normoxic conditions. Reoxygenation time varied depending on the experimental objectives: 2 h reoxygenation was performed for measurement of LDH release, whereas 6 h reoxygenation was performed for flow cytometry assay and measurements of cleaved caspase-3, Akt, and p-Akt expressions.

In the normal control group, cells were cultured with Tyrode's solution that contained 5.5 mmol/L glucose for 3 h and reoxygenated with DMEM for 2 h or 6 h. In the treatment groups, diltiazem (positive control, final concentration: 45 *μ*g/mL), citric acid, and L-malic acid (final concentrations: 200, 100, 50, 25 *μ*g/mL) were dissolved with dimethylsulfoxide (DMSO) and added, respectively, into the medium with the ratio of 1 : 1000 at the start of hypoxia and reoxygenation. For the normal control group and model control group, equivalent volumes of DMSO were added.

### 2.7. LDH Release

The hypoxia and reoxygenation supernatants were collected. After 2 h reoxygenation, cells were lysed by freeze thawing in distilled water. LDH activities were measured using the enzymatic reaction kinetics monitoring method according to the manufacturer's instructions.

The total LDH activity was obtained from adding LDH activities in the hypoxia and reoxygenation supernatants and the cell lysate together. The LDH release rate was calculated by dividing the sum of LDH activities in the hypoxia and reoxygenation supernatants into the total LDH activity. 

### 2.8. Flow Cytometry Analysis

Apoptosis was assessed by FITC-annexin V/propidium iodide apoptosis detection kit according to the manufacturer's protocol. Briefly, at the end of 6 h reoxygenation, cells were harvested with trypsin (0.25%) and centrifugation (1000 rpm for 5 min). Cells were washed twice with cold PBS and then resuspended in 200 *μ*L binding buffer at a concentration of 1 × 10^5^ cells/mL. Cells were incubated with 5 *μ*L FITC-annexin V and 5 *μ*L propidium iodide (PI) for 15 min in the dark at room temperature (25°C). Samples were analyzed by flow cytometry (Epics Elite, Beckman Coulter) immediately. Approximately 10 000 cells were counted for each sample and data were analyzed by using Expo32 software.

### 2.9. Western Blot Analysis

The expression levels of cleaved caspase-3, Akt, and p-Akt were measured by western blotting. Cells were washed with prewarmed PBS and then lysed at 4°C with ice-cold RIPA lysis buffer (50 mM Tris (pH 7.4), 150 mM NaCl, 1% Triton X-100, 1% sodium deoxycholate, 0.1% SDS, 1 mM PMSF, and phosphatase inhibitors mixture (#P1260, Applygen Technologies Inc.)) for 30 min. Cell lysates were then centrifuged at 12000 g at 4°C for 5 min and protein concentrations in the supernatants were determined by BCA protein assay kit (Beyotime Biotechnology). 

Samples with equivalent amounts of total protein (20 *μ*g) were loaded and separated by 10% SDS-polyacrylamide gel electrophoresis and transferred to polyvinylidene difluoride (PVDF) membranes (Bio-Rad). The membranes were blocked in 5% BSA for 1 h and then incubated overnight at 4°C with primary antibody (rabbit anti-cleaved caspase 3, rabbit anti-Akt, and rabbit anti-p-Akt at 1 : 500, 1 : 1000, and 1 : 2000 dilution, and mouse anti-*α*-actin (Beijing Biosynthesis Biotechnology, China) at 1 : 2000 dilution). The membranes were washed six times in 1 × Tris-buffer saline-Tween 20 (TBST) buffer and then incubated with horseradish-peroxidase-(HRP-) conjugated goat anti-rabbit or mouse secondary antibodies (1 : 40000 dilution) for 1 h at room temperature. After excess antibodies were removed by washing, bands were detected with an enhanced chemiluminescence (ECL) system (Thermo, USA) and visualized with the Chemi Doc XRS+ gel documentation system (Bio-Rad, USA) and analyzed by using Image lab 3.0 software (Bio-Rad, USA). The expression levels of *α*-actin served as an internal control for protein loading. 

### 2.10. Statistical Analysis

All data were presented as the mean ± SD. The data analyses were performed using one-way ANOVA analysis followed by Student-Newman-Keuls test for multiple comparisons. In all cases, values of *P* < 0.05 were considered statistically significant.

## 3. Results

### 3.1. Effects of Citric Acid and L-Malic Acid on Myocardial Infarct Size

**Figure 1:**
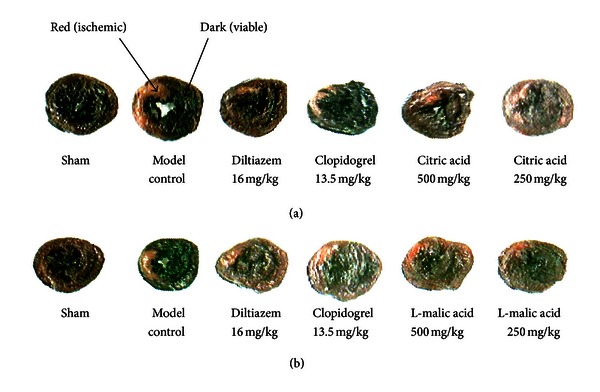
A representative N-BT staining of infarct size. The normal myocardium was stained dark, and the ischemic area was stained red. (a) Citric acid; (b) L-malic acid.

As illustrated in Figure 1(a) and Table 1, no myocardial infarction was observed in the sham group, while myocardial ischemia/reperfusion resulted in significant myocardial infarcts (*P* < 0.01). Diltiazem and clopidogrel, which were used as positive controls, significantly reduced infarction percentage of the ventricle, infarction percentage of the heart, infarction area, and infarction weight, as compared with the model control (*P* < 0.01 or *P* < 0.05). Compared with the model control group, treatments with citric acid at the doses of 500 mg/kg and 250 mg/kg significantly reduced infarction percentage of the ventricle, infarction percentage of the heart, infarction area, and infarction weight (*P* < 0.01 or *P* < 0.05).

A similar result was shown in Figure 1(b) and Table 2. Myocardial ischemia/reperfusion resulted in substantial myocardial infarcts, which were significantly reduced by treatments with diltiazem and clopidogrel (*P* < 0.01 or *P* < 0.05). Compared with the model control group, treatment with L-malic acid at the dose of 250 mg/kg significantly decreased infarction percentage of the ventricle, infarction percentage of the heart, infarction area, and infarction weight (*P* < 0.01), and treatment with L-malic acid at the dose of 500 mg/kg significantly decreased infarction percentage of the ventricle and infarction area (*P* < 0.01 and *P* < 0.05, resp.) but had only a tendency to reduce infarction percentage of the heart and infarction weight (*P* = 0.056 and *P* = 0.095, resp.). 

### 3.2. Effects of Citric Acid and L-Malic Acid on TNF-**α** Production following Myocardial Ischemia/Reperfusion

**Figure 2:**
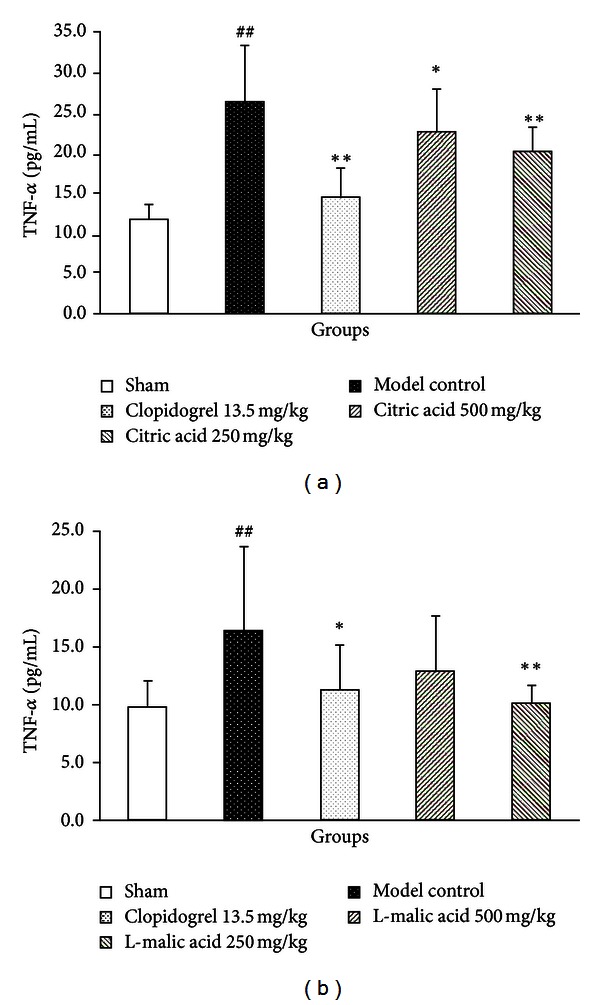
Effects of citric acid (a) and L-malic acid (b) on serum levels of TNF-**α** following myocardial ischemia/reperfusion. Data are shown as mean ± SD. ^##^ *P* < 0.01 versus sham, **P < 0.01, *P < 0.05 versus model control (*n* = 10).

Figure 2(a) showed that myocardial ischemia/reperfusion injury significantly increased the level of serum TNF-**α** compared with the sham group (26.71 ± 6.44 versus 11.84 ± 1.67 pg/mL, *P* < 0.05). Compared with the model control group, pretreatment with clopidogrel significantly reduced serum TNF-**α** level by 45.7% (14.51 ± 3.02 pg/mL, *P* < 0.01), and pretreatments with citric acid at the doses of 500 mg/kg and 250 mg/kg significantly reduced serum TNF-**α** levels by 15.2% (22.66 ± 5.22 pg/mL, *P* < 0.05) and 23.3% (20.49 ± 2.71 pg/mL, *P* < 0.01), respectively.

Similarly, Figure 2(b) showed that the level of serum TNF-**α** in the model control group was significantly increased (16.42 ± 7.27 versus 9.85 ± 2.25 pg/mL in the sham group, *P* < 0.05). Compared with the model control group, pretreatment with clopidogrel reduced serum TNF-**α** level by 30.8% (11.36 ± 3.73 pg/mL, *P* < 0.05), and pretreatment with L-malic acid at the dose of 500 mg/kg had only a tendency to decrease serum TNF-**α** level (12.98 ± 4.63 pg/mL, *P* = 0.129), while pretreatment with L-malic acid at the dose of 250 mg/kg significantly reduced serum TNF-**α** level by 37.9% (10.20 ± 1.50 pg/mL, *P* < 0.01).

### 3.3. Effects of Citric Acid and L-Malic Acid on Platelet Aggregation Induced by ADP following Myocardial Ischemia/Reperfusion

**Figure 3:**
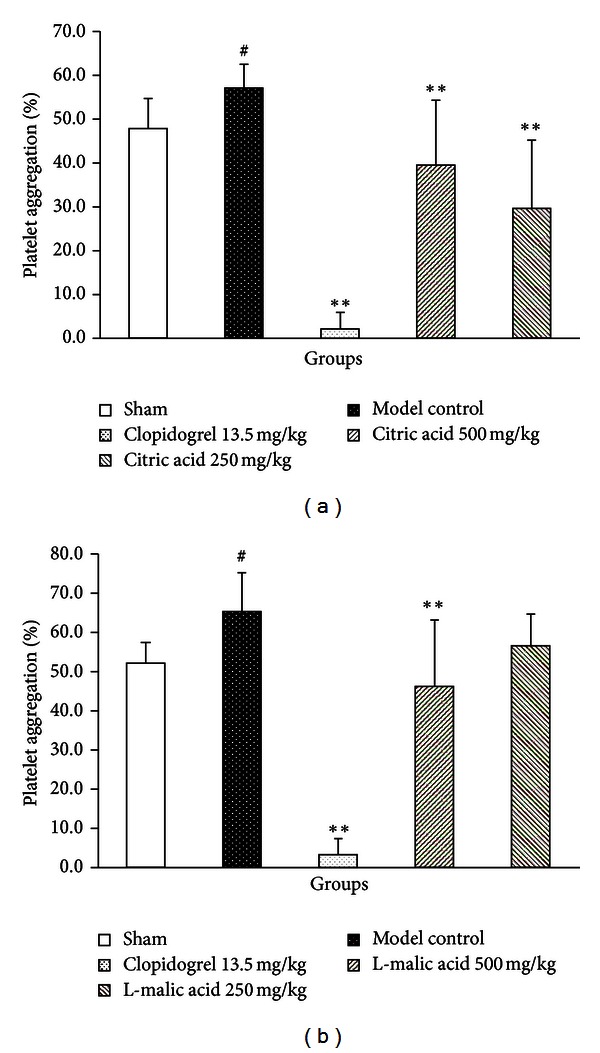
Effects of citric acid (a) and L-malic acid (b) on platelet aggregation induced by ADP following myocardial ischemi-reperfusion. Data are shown as mean ± SD. ^#^ *P* < 0.05 versus sham, **P < 0.01 versus model control (*n* = 10).

We measured the effects of citric acid and L-malic acid on platelet aggregation induced by one of the classical endogenous agonists ADP. As shown in Figure 3(a), compared with the sham group, myocardial ischemia/reperfusion significantly increased platelet aggregation rate induced by ADP (57.53 ± 7.47% versus 46.81 ± 6.18%, *P* < 0.05). Compared with the model control group, pretreatment with clopidogrel significantly reduced platelet aggregation rate (2.12 ± 3.44%, *P* < 0.01), and pretreatments with citric acid at the doses of 500 mg/kg and 250 mg/kg significantly decreased platelet aggregation rate (35.19 ± 13.29% and 27.50 ± 14.08%, resp., *P* < 0.01 each).

 A similar result was shown in Figure 3(b), the platelet aggregation rate in the model control group was significantly increased (66.43 ± 8.66% versus 53.06 ± 5.27% in the sham group, *P* < 0.05). Compared with the model control group, the platelet aggregation rate for the clopidogrel group was 3.36 ± 4.15% (*P* < 0.01), and that for L-malic acid at the doses of 500 mg/kg and 250 mg/kg groups was 47.02 ± 17.09% (*P* < 0.01) and 57.58 ± 8.09% (*P* = 0.149), respectively.

### 3.4. Effects of Citric Acid and L-Malic Acid on H/R-Induced Cardiomyocyte Necrosis

**Figure 4:**
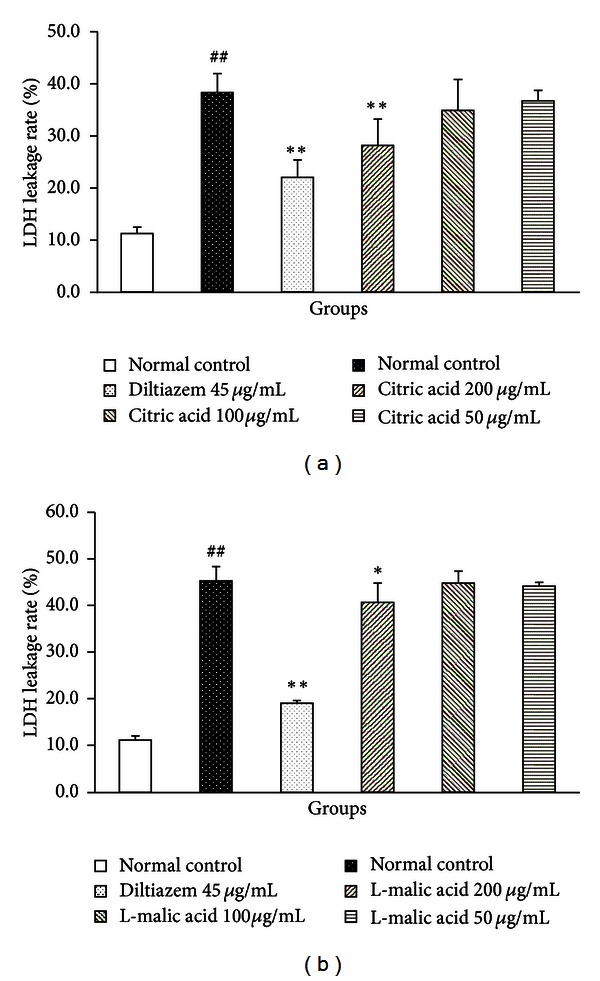
Effects of citric acid (a) and L-malic acid (b) on LDH release. Cardiomyocytes were subjected to 3 h hypoxia followed by 2 h reoxygenation with or without treatment. LDH activities in the hypoxia and the reoxygenation media and in the cell lysates were measured. Data are shown as mean ± SD. ^##^ *P* < 0.01 versus normal control, **P < 0.01,*P < 0.05 versus model control (*n* = 3).

LDH leakage from cells was widely used as a reliable marker of cellular injury. The degree of LDH release was closely related to cardiomyocyte necrosis [16, 17]. Thus, we explored the protective effects of citric acid and L-malic acid on H/R-induced cardiomyocyte injury *in vitro* by detecting LDH release.  Figure 4(a) showed that after cardiomyocytes were subjected to 3 h hypoxia followed by 2 h reoxygenation, a significant LDH release was induced (40.76 ± 2.88% versus 14.57 ± 0.96% in the normal control group, *P* < 0.01), which were significantly inhibited by diltiazem (23.25 ± 2.61%, *P* < 0.01) and citric acid at the concentration of 200 *μ*g/mL (31.07 ± 5.54%, *P* < 0.01).

A similar result was shown in Figure 4(b), the LDH release rate in the model control group was significantly increased (45.31 ± 3.00% versus 11.23 ± 0.86% in normal control group, *P* < 0.01). Compared with the model control group, the LDH release rate for diltiazem group was 19.12 ± 0.57% (*P* < 0.01), and that for L-malic acid at the concentration of 200 *μ*g/mL group was 40.69 ± 4.03% (*P* < 0.05).

### 3.5. Effects of Citric Acid and L-Malic Acid on H/R-Induced Cardiomyocyte Apoptosis

**Figure 5:**
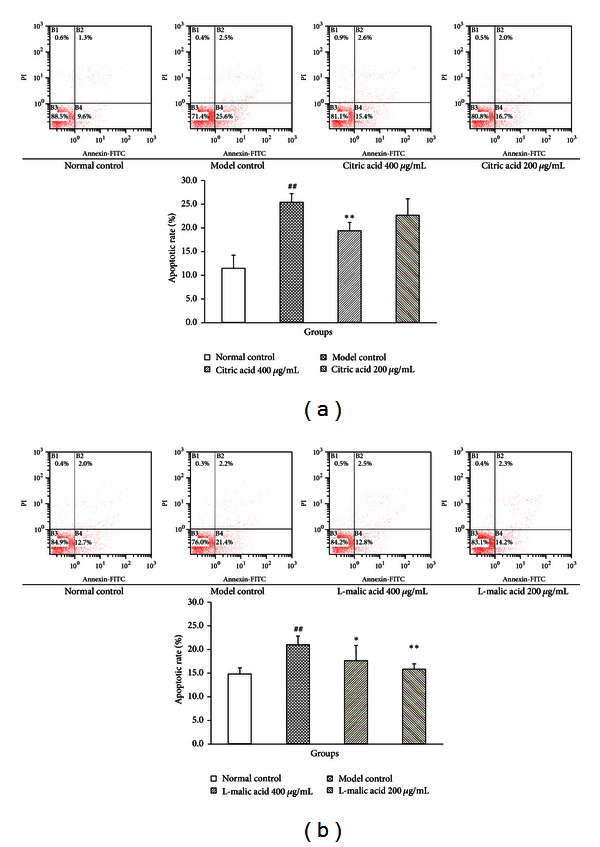
Effects of citric acid (a) and L-malic acid (b) on H/R-induced cardiomyocyte apoptosis. Cardiomyocytes were subjected to 3 h hypoxia and 6 h reoxygenation in the presence or absence of citric acid or L-malic acid. Data are shown as mean ± SD. ^##^ *P* < 0.01 versus normal control, **P < 0.01, *P < 0.05 versus model control (*n* = 3).

Based on the previous results that treatments with citric acid and L-malic acid below concentration of 200 *μ*g/mL could not decrease LDH release, we chose to use citric acid and L-malic acid at concentrations of 400 *μ*g/mL and 200 *μ*g/mL to observe whether citric acid and L-malic acid could decrease H/R-induced cardiomyocyte apoptosis. As data shown in Figure 5(a), after cardiomyocytes were subjected to 3 h hypoxia followed by 6 h reoxygenation injury, the number of apoptotic cells was significantly increased as compared with the normal control group (25.45 ± 1.81% versus 11.48 ± 2.74%, *P* < 0.01). In contrast, treatments with citric acid at concentrations of 400 *μ*g/mL and 200 *μ*g/mL reduced the number of apoptotic cells to 19.43 ± 1.69% (*P* < 0.01) and 22.70 ± 3.47% (*P* = 0.179), respectively.

Similarly, Figure 5(b) showed that the number of apoptotic cells in the model control group was significantly increased (22.13 ± 1.69% versus 15.65 ± 1.34% in the normal control group, *P* < 0.01), which was significantly reduced by treatments with L-malic acid at concentrations of 400 *μ*g/mL (18.63 ± 3.17%, *P* < 0.05) and 200 *μ*g/mL (16.70 ± 0.62%, *P* < 0.01), respectively.

### 3.6. Effects of Citric Acid and L-Malic Acid on Expression of Cleaved Caspase-3

**Figure 6:**
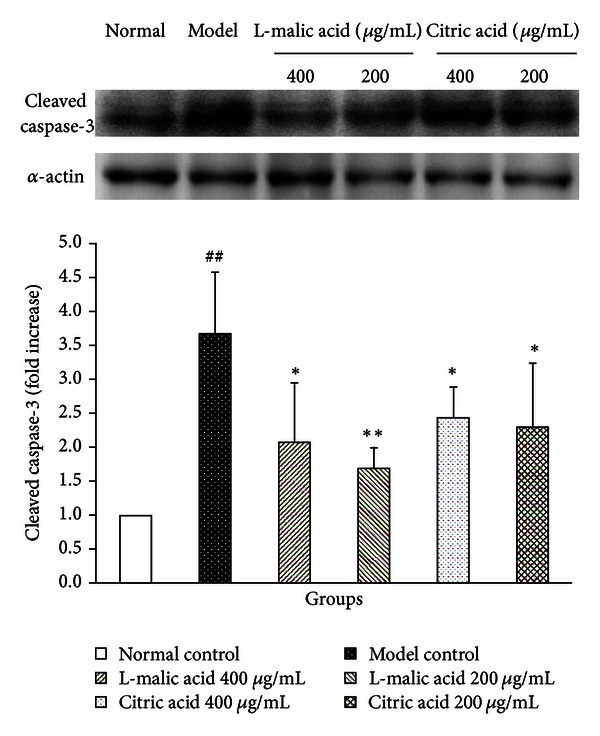
Effects of citric acid and L-malic acid on expression levels of cleaved caspase-3 (fold increase relative to normal control levels) after cardiomyocytes subjected to 3 h hypoxia followed by 6 h reoxygenation. Data are shown as mean ± SD. ^##^ *P* < 0.01 versus normal control, **P < 0.01, *P < 0.05 versus model control. Results are representative of three independent experiments.

Next we investigated the effects of citric acid and L-malic acid on expression levels of cleaved caspase-3, the activated form of caspase-3. As shown in Figure 6, western blot analysis revealed that the expression of cleaved caspase-3 was significantly upregulated (2.69-fold, *P* < 0.01) after cardiomyocytes were subjected to 3 h hypoxia followed by 6 h reoxygenation, which was significantly downregulated by treatments with citric acid at the concentrations of 400 *μ*g/mL (33.60%, *P* < 0.05) and 200 *μ*g/mL (37.40%, *P* < 0.05) and treatments with L-malic acid at concentrations of 400 *μ*g/mL (43.63%, *P* < 0.05) and 200 *μ*g/mL (53.93%, *P* < 0.01), respectively.

### 3.7. Effects of Citric Acid and L-Malic Acid on Expression Levels of Akt and p-Akt

**Figure 7:**
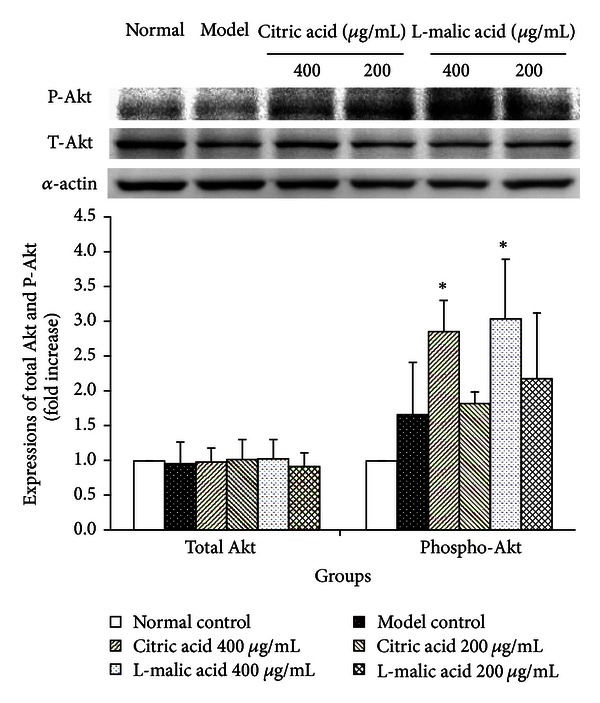
Citric acid and L-malic acid activate PI/3K and phosphorylation of Akt. A representative western blot analysis of total Akt and phosphorylation of Akt at Ser473 after cardiomyocytes were subjected to 3 h hypoxia followed by 6 h reoxygenation. Data are shown as mean ± SD. *P < 0.05 versus model control. Results are representative of three independent experiments.

The PI3 K/Akt pathway plays a critical role in survival after myocardial ischemia/reperfusion injury. Phosphorylation of Akt S473 represents its maximal activation [18, 19]. To determine whether Akt was involved in citric acid and L-malic acid protection from cardiomyocyte injury, we further detected the expressions of Akt and phospho-Akt (Ser 473). As illustrated in Figure 7, western blotting results showed that total Akt was comparable in all groups. The densities of phosphorylated Akt were normalized against total Akt. We found that H/R-induced cardiomyocyte injury by itself resulted in a 0.67-fold increase in Akt phosphorylation, while treatments with both citric acid at the concentration of 400 *μ*g/mL and L-malic acid at the concentration of 400 *μ*g/mL further significantly upregulated the expression levels of phosphorylated Akt after cardiomyocytes hypoxia/reoxygenation injury compared with the model control group (0.71-fold and 0.82-fold, resp., *P* < 0.05 each). Treatments with both citric acid at the concentration of 200 *μ*g/mL and L-malic acid at the concentration of 200 *μ*g/mL had a tendency to increase the expression levels of phosphorylated Akt (9.0% and 30.54%, resp., *P* = 0.763 and *P* = 0.337, resp.), without significant differences.

## 4. Discussion

In the present study, we reported for the first time the *in vivo* data demonstrating that pretreatments with both citric acid and L-malic acid significantly ameliorated the I/R-induced cardiac injury, including reduced myocardial infarct size, decreased inflammatory cytokine TNF*-*α** activity, and inhibited ADP-induced platelet aggregation. Furthermore, *in vitro* experiments revealed that both citric acid and L-malic acid protected cardiomyocyte damage from necrosis and apoptosis during cardiomyocyte hypoxia/reoxygenation injury possibly via a mechanism involving PI3K/Akt survival pathway. 

In recent years, traditional Chinese medicine has been greatly developed in many countries due to its high quality and safety [20–22], and considerable attention has focused on the material basis of Chinese medicine studies. The material basis of *Fructus Choerospondiatis* responsible for its cardioprotective effects has been always considered to be flavonoids (mainly quercetin). However, our data *in vivo* demonstrated that both citric acid (500, 250 mg/kg) and quercetin (40, 20 mg/kg) significantly ameliorated the I/R-induced cardiac injury (data unpublished), and the *in vitro* experiments confirmed that both organic acids (citric acid, L-malic acid, succinic acid, and tartaric acid; concentration: 400 *μ*g/mL) and flavonoids (quercetin and kaempferol; concentrations: 12.5, 25, 50, and 100 *μ*g/mL) significantly decreased LDH release rate of cardiomyocytes injured by hypoxia/reoxygenation [23, 24]. Although the dosage of organic acids used in these studies was 10~30 times higher than that of flavonoids, the content of total organic acids in *Fructus Choerospondiatis* was nearly 30 000 times higher than that of total flavonoids. Therefore, our results still furnish strong evidence that organic acids may also be the major active ingredients of *Fructus Choerospondiatis* responsible for its cardioprotective effects.

The extent of myocardial damage is closely related to prognosis. Therefore, determination of infarct size is the strongest determinant of prognosis of ischemic heart disease [25]. The results showed that pretreatments with both citric acid and L-malic acid significantly reduced I/R-induced myocardial infarct size and thus protected the infarcted myocardium.

Inflammatory responses and platelet aggregation have been implicated in myocardial ischemia/reperfusion injury [26]. Within minutes after reperfusion, inflammatory cascade is triggered and copious amounts of proinflammatory cytokines such as TNF*-*α**, IL-1*β*, IL-6, and IL-8 are produced and released [27]. These proinflammatory cytokines (particularly TNF*-*α**), as important factors in cardiac dysfunction, activate neutrophils and endothelial cells and aggravate myocardial ischemia/reperfusion injury [28, 29]. Platelets play a critical role in the process of myocardial ischemia/reperfusion injury. After reperfusion, platelets are immediately activated and increased, and platelet aggregability will aggravate myocardial ischemia/reperfusion injury in turn, which may be related to endothelial dysfunction and platelet-derived p-selectin, and so forth [30]. The results showed that both citric acid and L-malic acid decreased TNF*-*α** level and inhibited platelet aggregation on myocardial ischemia/reperfusion injury. These data *in vivo* provided direct evidence that organic acids protected ischemia myocardium may be partly due to inhibition of inflammation and platelet aggregation. 

Cardiomyocyte necrosis and apoptosis are the major contributors to myocardial ischemia/reperfusion injury [31]. Cardiomyocyte loss, caused by both necrosis and apoptosis, is the main feature of myocardial ischemia/reperfusion injury [32]. Necrosis and apoptosis are two distinct types of cell death with different characteristics. Necrosis leads to membrane lysis, release of cellular contents, and resulting inflammation, while apoptosis is characterized by cell shrinkage, membrane blebbing, and nuclear condensation and degradation [33]. Necrotic cells are mainly found in the central zone of the infarct, while apoptotic cells are more apparent at the marginal zone [33]. It may be beneficial for attenuating necrosis and apoptosis to prevent cardiomyocyte loss caused by myocardial ischemia/reperfusion injury. Thus, after an initial investigation of the effects of citric acid and L-malic acid on myocardial ischemia/reperfusion injury in *in vivo* rat model, we further observed their cardioprotective effects in cellular level. The concentrations of citric acid and L-malic acid (400 *μ*g/mL and 200 *μ*g/mL) used in this study had been evaluated on cytotoxicity, as determined by MTT assay. There were no significant differences between citric acid or L-malic acid at the concentrations below 500 *μ*g/mL and the control group [23]. LDH is a stable cytosolic enzyme present in mammalian cells and LDH release is an indication of cell membrane integrity. The amount of LDH released from cells is proportional to the extent of membrane damage and cell necrosis [34]. The data showed that H/R injury induced significant LDH release, but treatments with citric acid at the concentration of 200 *μ*g/mL and L-malic acid at the concentration of 200 *μ*g/mL could significantly reduce cardiomyocyte LDH release rate.

Furthermore, we studied the effects of citric acid and L-malic acid on hypoxia/reoxygenation-induced apoptosis by flow cytometry analysis. The results showed that H/R injury significantly increased the number of apoptotic cardiomyocytes, while treatments with citric acid at the concentration of 400 *μ*g/mL or L-malic acid at the concentrations of 400 *μ*g/mL and 200 *μ*g/mL significantly reduced the number of apoptotic cells. The cleavage of caspase-3 is often identified as the important step in the apoptotic signaling pathway activation process and it is considered to be a potential molecular therapeutic target for preventing cardiomyocyte apoptosis [35]. We next investigated the expression of cleaved caspase-3 and found that the cleaved caspase-3 was significantly upregulated by hypoxia/reoxygenation-induced cardiomyocyte injury while significantly downregulated by treatments with citric acid at the concentrations of 400 *μ*g/mL and 200 *μ*g/mL or L-malic acid at the concentrations of 400 *μ*g/mL and 200 *μ*g/mL relative to the model control group.

Akt is a potent cell survival factor and an important downstream kinase of PI3 K. The phosphorylation and activation of Akt play a pivotal role in myocardial ischemia/reperfusion injury [36]. Considerable evidence suggests that the activation of Akt reduced myocardial infarct size [17, 37]. To investigate whether Akt is involved in citric acid and L-malic acid-induced cardioprotection, we evaluated the expression of Akt and its activated, phosphorylated form (phospho-Akt) at Ser473 after hypoxia/reoxygenation-induced cardiomyocyte injury. Our results showed that treatments with citric acid and L-malic acid significantly upregulated the expression of phosphorylated Akt. All data *in vitro* concluded that the cardioprotection of citric acid and L-malic acid contributed to preventing cardiomyocyte from necrosis and apoptosis, which may have the PI3K/Akt survival pathway involved.

In conclusion, the present study demonstrates that citric acid and L-malic acid have protective effects on myocardial ischemia/reperfusion injury; the underlying mechanism may be associated with their anti-inflammatory, anti-platelet aggregation and direct cardiomyocyte protective effects. Based on these findings, we concluded that organic acids may also be the major active ingredients of *Fructus Choerospondiatis* responsible for its cardioprotective effects but not only flavonoids now.

## Figures and Tables

**Table 1:** The effect of citric acid on myocardial ischemia/reperfusion injury in rats ($\bar{x}\pm s$, *n *= 10).

Groups	Dosage (mg/kg)	Infarction of the ventricle (%)	Infarction of the heart (%)	Infarction area (mm^2^)	Infarction weight (g)
Sham	—	0.00 ± 0.00	0.00 ± 0.00	0.00 ± 0.00	0.000 ± 0.000
Model control	—	17.30 ± 3.58^##^	11.98 ± 2.20^##^	49.75 ± 9.14^##^	0.086 ± 0.017^##^
Diltiazem	16	8.23 ± 2.50**	5.33 ± 1.11**	21.90 ± 4.78**	0.038 ± 0.007**
Clopidogrel	13.5	11.35 ± 2.71**	8.52 ± 1.77**	35.17 ± 9.74**	0.064 ± 0.015*
Citric acid	500	12.16 ± 4.27**	8.80 ± 3.41**	36.18 ± 15.23**	0.064 ± 0.026*
	250	11.80 ± 3.67**	8.83 ± 3.09**	33.97 ± 10.99**	0.063 ± 0.022**

^##^ *P* < 0.01 versus sham, **P < 0.01, *P < 0.05 versus model control.

**Table 2:** The effect of L-malic acid on myocardial ischemia/reperfusion injury in rats ($\bar{x}\pm s$, *n* = 10).

Groups	Dosage (mg/kg)	Infarction of the ventricle (%)	Infarction of the heart (%)	Infarction area (mm^2^)	Infarction weight (g)
Sham	—	0.00 ± 0.00	0.00 ± 0.00	0.00 ± 0.00	0.000 ± 0.000
Model control	—	9.03 ± 3.32^##^	6.00 ± 1.67^##^	32.01 ± 11.84^##^	0.051 ± 0.014^##^
Diltiazem	16	6.86 ± 1.76**	4.68 ± 1.39*	25.11 ± 6.48*	0.041 ± 0.012*
Clopidogrel	13.5	6.51 ± 1.58**	4.75 ± 1.19*	23.67 ± 5.46*	0.038 ± 0.010*
L-malic acid	500	6.71 ± 1.23**	4.98 ± 0.92	25.37 ± 4.55*	0.043 ± 0.009
	250	6.22 ± 1.15**	4.48 ± 0.93**	22.70 ± 4.26**	0.036 ± 0.007**

^##^ *P* < 0.01 versus sham, **P < 0.01, *P < 0.05 versus model control.
